# Unilateral Left Ankyloblepharon Filiforme Adnatum Associated With Trisomy 18 in a Term Female Neonate: A Case Report

**DOI:** 10.7759/cureus.113402

**Published:** 2026-07-26

**Authors:** Hanae Mezrhab, Mohammed Ech-Chebab, Anass Ayyad, Sahar Messaoudi, Rim Amrani

**Affiliations:** 1 Pediatrics, Mohammed VI University Hospital Center, Oujda, MAR; 2 Neonatology and Neonatal Intensive Care, Mohammed VI University Hospital Center, Oujda, MAR; 3 Neonatology and Neonatal Resuscitation, Faculty of Medicine and Pharmacy, Mohamed First University of Oujda, Oujda, MAR; 4 Neonatology, Mohammed VI University Hospital Center, Oujda, MAR; 5 Faculty of Medicine and Pharmacy of Oujda, Mother and Child Health Laboratory, Oujda, MAR

**Keywords:** ankyloblepharon filiforme adnatum, case report, congenital eyelid anomaly, edwards syndrome, neonate, trisomy 18

## Abstract

Ankyloblepharon filiforme adnatum (AFA) is a rare, congenital eyelid anomaly characterized by partial fusion of the upper and lower eyelid margins by fine extensile tissue bands. We report a term female neonate with unilateral left AFA, intrauterine growth restriction, dysmorphic features, and congenital heart disease. Clinical evaluation raised suspicion of trisomy 18, which was confirmed by blood karyotyping. Echocardiography revealed a complex congenital heart defect suggestive of tetralogy of Fallot. The eyelid band was excised with scissors without sedation or local anesthesia, resulting in immediate restoration of palpebral opening and clearance of the visual axis without bleeding. This case highlights the importance of early treatment of AFA and systematic evaluation for associated malformations and chromosomal abnormalities.

## Introduction

Ankyloblepharon filiforme adnatum (AFA) is a rare, congenital eyelid anomaly in which eyelid development is complete, but the separation of the eyelids remains incomplete at birth [1]. Clinically, it presents as one or more fine, extensile bands joining the upper and lower eyelid margins and may occur either unilaterally or bilaterally [2,3]. Although AFA is often an isolated and benign finding, its presence should alert the clinician to possible ocular, systemic, and syndromic associations [4-6].

Early diagnosis is critical because the obstruction of the visual axis may lead to stimulus deprivation amblyopia if treatment is delayed [2,6]. Previous reports have described associations with ectodermal syndromes, cleft lip and palate, curly hair-ankyloblepharon-nail disease (CHAND) syndrome, cardiac defects, and chromosomal abnormalities such as trisomy 18 [1,3]. Clark and Patterson specifically reported AFA in Edwards syndrome, and subsequent reviews have consistently classified trisomy 18 among the syndromic associations of AFA [1,7]. Because AFA is exceptionally rare in the context of trisomy 18, documenting these cases is vital for improving early clinical recognition of underlying chromosomal defects.

The objective of this case report is to highlight a rare presentation of unilateral AFA as an early clinical indicator of trisomy 18. By detailing the clinical, radiological, and genetic findings of this case, we aim to emphasize the necessity of comprehensive systemic evaluation in neonates presenting with AFA.

## Case presentation

A female neonate was admitted in July 2023 to the neonatal intensive care unit on her first day of life. She was born at term, at 39 weeks of gestation, to a 21-year-old mother G1P1 with hypothyroidism managed with levothyroxine. There was no family history of congenital anomalies and no parental consanguinity. Delivery was performed by cesarean section due to prenatal ultrasonographic suspicion of congenital heart disease and severe intrauterine growth restriction.

At birth, the neonate exhibited profound growth restriction: her weight was 1400 g, length was 42 cm, and head circumference was 28 cm (all parameters below the 3rd percentile). A detailed pediatric examination revealed prominent dysmorphic craniofacial features, including microcephaly with a narrow, dolichocephalic skull, microretrognathia, hypertelorism, and fine facial features. These dysmorphic traits immediately raised clinical suspicion for a chromosomal abnormality, specifically trisomy 18. Cardiovascular auscultation disclosed a distinct systolic murmur.

**Figure 1:**
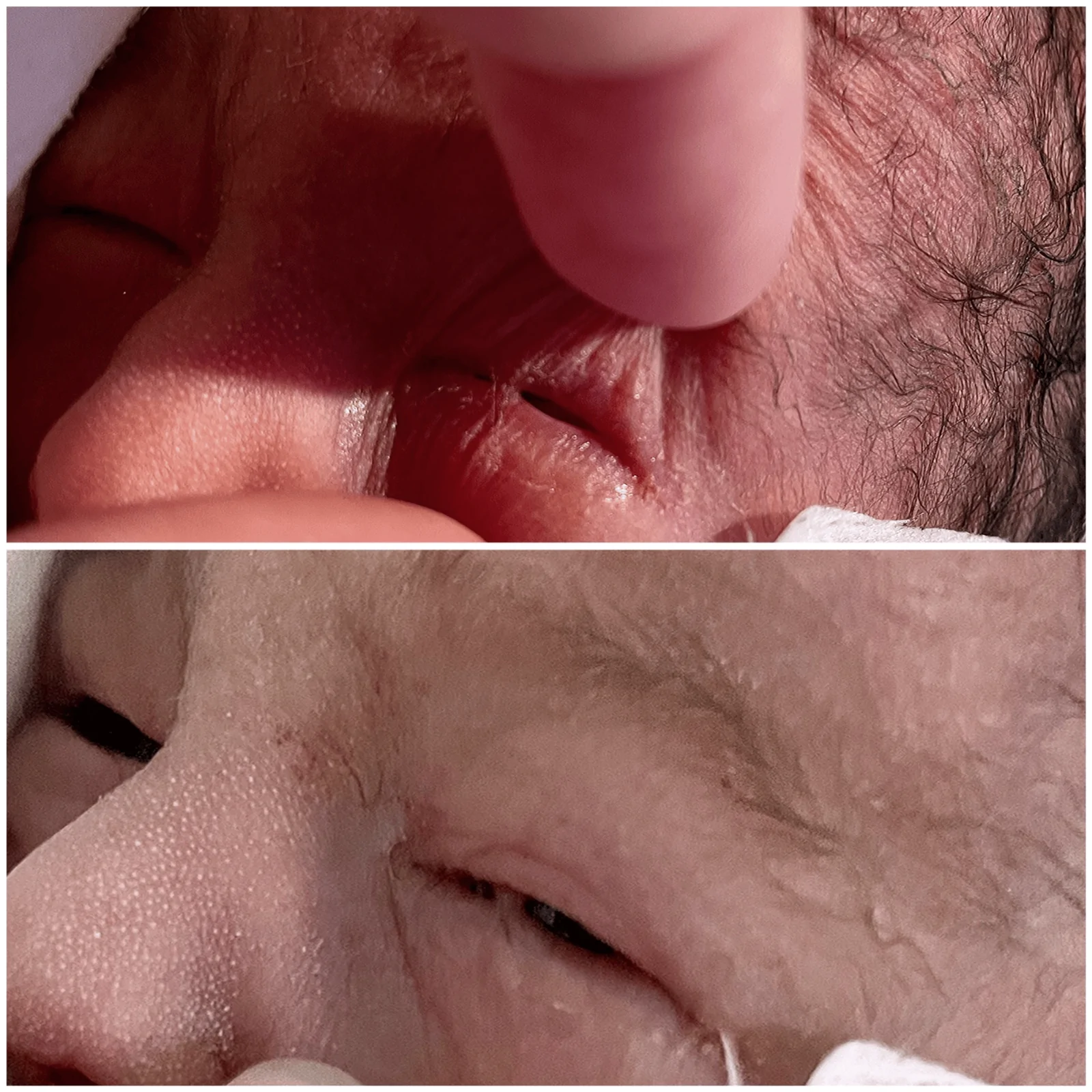
Clinical photograph demonstrating ankyloblepharon filiforme adnatum (AFA) of the left eye prior to intervention A fine, extensile tissue band partially fuses the upper and lower eyelid margins, obstructing the visual axis.

An initial ophthalmologic examination revealed unilateral involvement of the left eye, characterized by a partial fusion of the left upper and lower eyelids by a thin, fibrous band of tissue, consistent with ankyloblepharon filiforme adnatum (Figure 1). No similar lesions or other ocular abnormalities were present in the right eye.

A comprehensive malformation workup was initiated to evaluate the suspected syndromic presentation. Echocardiography demonstrated a malaligned ventricular septal defect with an overriding aorta, moderate valvular pulmonary stenosis, and a 1-mm patent ductus arteriosus. This structural constellation was suggestive of tetralogy of Fallot. To confirm the genetic etiology of the severe growth restriction, dysmorphic features, and cardiac anomalies, blood samples were sent for karyotyping. The cytogenetic analysis revealed a 47,XX,+18 karyotype, definitively confirming the diagnosis of trisomy 18 (Edwards syndrome). Standard laboratory evaluations, including a complete blood count and metabolic panel, were unremarkable. Radiological evaluations of the abdomen and renal system, apart from a transfontanellar ultrasound, showed no further malformations.

**Figure 2:**
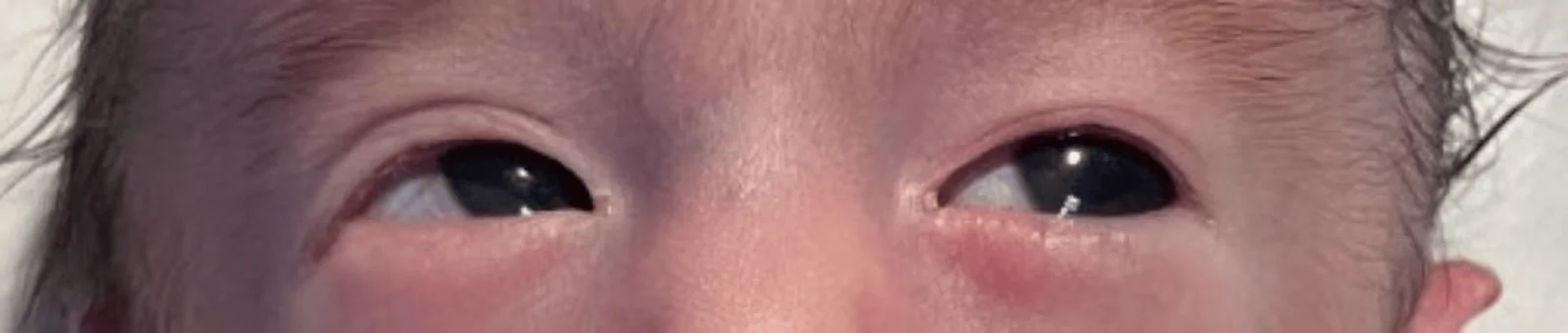
Clinical photograph of the left eye immediately following surgical incision of the fibrous band There is complete restoration of the palpebral opening and visual axis clearance with no associated bleeding.

Given the risk of occlusion amblyopia, the fibrous eyelid band was excised with sterile Westcott scissors at the eyelid margin of the left eye (Figure 2). The procedure was performed at the bedside without sedation or local anesthesia. No bleeding or complications occurred. The intervention achieved an immediate improvement in palpebral opening, allowing complete clearance of the visual axis in primary gaze and facilitating a full posterior segment examination, which was normal.

Regarding follow-up, the patient’s cardiopulmonary status remained highly fragile during her stay in the neonatal intensive care unit due to the complex congenital heart defect and the underlying chromosomal syndrome. Unfortunately, her clinical condition rapidly deteriorated, and she died prior to the realization of any definitive cardiac or systemic surgical procedure.

## Discussion

The present case is notable for the strictly unilateral involvement of the left eye and the severe syndromic context in which the lesion occurred. When comparing our findings with the existing literature, a significant divergence in prognosis and clinical associations becomes apparent. A substantial portion of the documented AFA cases describes isolated lesions in otherwise healthy newborns. In these isolated cases, the anomaly is localized, and simple excision results in highly favorable functional and cosmetic outcomes with excellent long-term prognoses [4,5]. Conversely, AFA can also occur as part of a broader malformation syndrome, most notably trisomy 18. The association between AFA and trisomy 18 was first reported by Clark and Patterson in 1985 in a female neonate who presented with bilateral eyelid bands, micrognathia, a high-arched palate, and characteristic limb abnormalities [7]. Tüysüz et al. described another female neonate presenting with AFA and trisomy 18, who also exhibited overlapping features of Hay-Wells syndrome (ectodermal dysplasia), severe cardiac malformations, and distinct craniofacial signs [8]. While the ocular presentation in our patient is a fine, fibrous band treatable by bedside excision that is morphologically identical to the bands seen in isolated AFA [2,6], our patient's systemic context aligns closely with the severe, multi-system disruptions observed by both Clark and Patterson and Tüysüz et al. [7,8].

The logical reasoning for these distinct clinical trajectories lies in their underlying embryological pathophysiology. Isolated AFA is thought to represent a localized failure of apoptosis during the normal separation of the eyelids between the ninth and twenty-eighth weeks of gestation [1]. In contrast, when AFA is associated with trisomy 18, it is not merely a local apoptotic failure but rather a superficial manifestation of a profound, global disruption of embryogenesis [8]. As postulated by Tüysüz et al., trisomy 18 causes abnormal cellular proliferation and hyperplasia that disrupts tissue fusion mechanisms at multiple sites simultaneously [8]. This widespread developmental impairment explains the severe concurrent anomalies such as the tetralogy of Fallot and profound intrauterine growth restriction observed in our patient and others [7,8].

Consequently, the striking visual presence of the eyelid anomaly may overshadow subtler, yet life-threatening, systemic disorders. If AFA is treated in isolation, without pursuing a complete malformation workup, critical diagnoses could be delayed. While the visual axis was restored successfully in our patient, her overall prognosis was fundamentally dictated by the severe systemic burdens associated with trisomy 18, ultimately leading to her early death.

Therefore, we strongly recommend that AFA should never be regarded as a purely local eyelid abnormality. All neonates presenting with AFA must undergo an immediate, comprehensive systemic, cardiological, and genetic evaluation to rule out life-threatening malformations before finalizing their care plan.

## Conclusions

Ankyloblepharon filiforme adnatum is an uncommon congenital eyelid anomaly that can occur in isolation or as part of a complex syndrome. This case report illustrates a rare presentation of unilateral AFA as the presenting ocular sign of trisomy 18 in a term neonate with severe intrauterine growth restriction and congenital heart disease. Ultimately, the ocular anomaly served as the primary clinical clue to a broader syndromic diagnosis.
